# Implementation of High-Dose-Rate, CT-Based Prostate Brachytherapy in an Academic Teaching Hospital and Residency Training Program

**DOI:** 10.7759/cureus.22494

**Published:** 2022-02-22

**Authors:** Daniel Brunnhoelzl, Alexander Hanania, Sam Sun, Sergio Jaramillo, Linfeng Lu, Pavan Jhaveri

**Affiliations:** 1 Radiation Oncology, Dan L. Duncan Comprehensive Cancer Center Baylor College of Medicine, Houston, USA; 2 Radiation Oncology, Covenant Medical Group, Lubbock, USA; 3 Radiation Oncology, Willis-Knighton Cancer Center, Shreveport, USA

**Keywords:** radiation oncology education, general radiation oncology, high dose-rate (hdr) brachytherapy, ct-based prostate brachytherapy, prostate brachytherapy, prostate cancer

## Abstract

Introduction

Prostate brachytherapy provides the most durable local control for prostate adenocarcinoma among all radiation treatment options. However, likely due to decreased trainee familiarity with the technique and resource intensity, it has seen a decline in utilization. Here we outline our experience with starting a high-dose-rate (HDR) prostate brachytherapy program within a residency training program and present our outcome data.

Methods

Patients were identified and screened using clinical data and volume study for candidacy for brachytherapy implantation. Eligible candidates were implanted and subsequently had radiation planning and delivery in our clinic. Descriptive statistical analysis was performed on our outcomes and dosimetry data and presented in tabular form.

Results

Seventeen patients were treated for a total of 18 implants (one monotherapy). No implant was aborted. No acute urinary retention requiring catheterization or chronic urethral stricture occurred. Biochemical recurrence-free survival was 94% at a median follow-up of 28.5 months (range 8.2-50 months); the one failure occurred in a very high-risk patient at 37 months following treatment. Dosimetrically, prostate coverage, urethra sparing, and rectum sparing aims were met. Volumetric bladder aims were also met; however, the max point dose to the bladder neck was above the guideline.

Conclusion

Our department successfully implemented an HDR prostate brachytherapy program. Treatments were effective and there was no grade 3 toxicity to report.

## Introduction

Brachytherapy (BT) is among the oldest and most cost-effective treatments for prostate cancer. It provides durable local control while maintaining high levels of patient satisfaction in terms of convenience, outcomes, and quality of life [1]. Prospective, randomized evidence from ASCENDE-RT (Androgen Suppression Combined with Elective Nodal and Dose Escalated Radiation Therapy) revealed that patients receiving dose-escalated external beam radiation therapy (EBRT) are twice as likely to experience biochemical failure compared to patients undergoing EBRT with a low dose rate (LDR) BT boost [2-5].

Due in part to the high level of evidence from ASCENDE-RT, the American Society of Clinical Oncology (ASCO)/Cancer Care Ontario (CCO) Joint Guideline Update published in 2017 states that for patients with high-risk prostate cancer receiving EBRT and androgen deprivation therapy (ADT), BT boost (either LDR or HDR) should be offered to eligible patients [6].

Unfortunately, trainee exposure to this modality may be a barrier to incorporation in future practice; in a survey conducted by Marcrom et al., most senior residents (postgraduate year (PGY) IV/V) respondents (59%) believed that caseload was the greatest barrier to achieving independence in BT. Only 24% (21 of 89) felt the five-case minimum for interstitial BT was adequate. Furthermore, there was an association between the aggregate number of BT cases performed and resident confidence in starting a BT practice (τ = 0.37; P < .001) [7].

A National Cancer Database (NCDB) study of over 100,000 men with intermediate and high-risk cancers revealed a concerning decline in the utilization of BT boost; for intermediate-risk patients, use decreased from 33.1% in 2004 to 12.5% in 2013, and for high-risk patients, utilization dropped from 27.6% to just 10.8% [8].

With the overall decline in BT utilization and concerns about trainee caseload [7-9], building a prostate BT program should be of priority to residency programs. Therefore, there is an urgent, unmet need for more exposure to this effective modality among radiation oncology trainees to promote adoption into future practices, as well as an unmet need for increased availability of this treatment in existing clinical practice nationwide, particularly for patients with unfavorable intermediate and high-risk disease. It is the goal of this paper to provide a framework of our experience to help guide practices that are interested in adopting this modality to better care for their patient population, as well as present our outcomes and dosimetric data.

## Materials and methods

Patient selection

An HDR-BT boost can be considered for high-risk or unfavorable intermediate-risk prostate cancer patients according to NCCN guidelines and consensus statements. Anesthesia can be with general anesthesia, an epidural, or conscious sedation. For centers utilizing general anesthesia, patient selection must take into consideration anesthesia tolerance from a cardiopulmonary perspective. Another consideration is that while, generally, an epidural can be performed by a certified nurse anesthetist, they require oversite by an MD anesthesiologist. Therefore, an epidural with Certified Registered Nurse Anesthetists (CRNAs) may be optimal for radiation centers attached to hospitals or outpatient surgery centers. The following are relative contraindications to HDR-BT: (1) AUA > 16 due to the risk of late urinary toxicity including catheter dependence; (2) median lobe hypertrophy (MLH), as catheters would have to be advanced to cover median lobe leading to excessive bladder dose; (3) large prostate volume (typically >60 mL); (4) pubic arch interference (PAI), as anterior needle placement may be limited resulting in a risk of inadequate prostate coverage (this can be assessed by doing a volume study in the lithotomy position as below); (5) previous transurethral resection of the prostate (TURP); (6) inflammatory bowel disease; (7) prior prostate radiation. Cases referred for prostate radiotherapy were evaluated at a BT peer-review conference consisting of multiple radiation oncologists experienced with BT. The case review included a review of history and pertinent imaging with a final disposition to dose-escalated EBRT alone or pelvis EBRT with HDR-BT boost. For patients with unfavorable intermediate-risk or high-risk prostate cancer, we utilized intensity-modulated radiotherapy (IMRT) to a prescribed dose of 45 Gy in 25 fractions or 44 Gy in 22 fractions to prostate and seminal vesicles +/- elective pelvic nodes at the discretion of the treating radiation oncologist.

**Figure 1:**
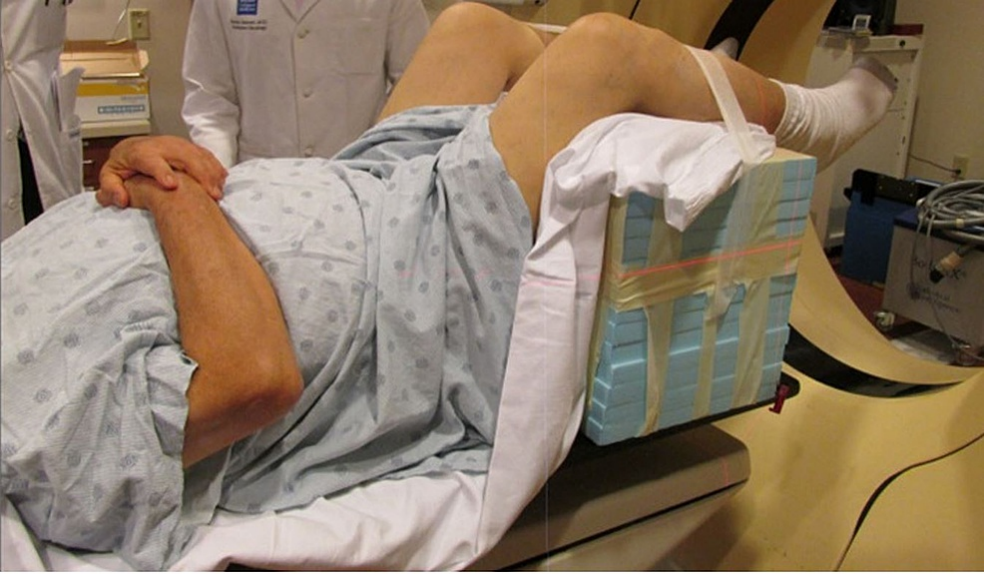
Patient in the lithotomy position for a volume study to assess candidacy for a prostate implant

Patients underwent CT simulation for EBRT, with bowel prep and a full bladder, as well as the CIVCO wing board (Coralville, Iowa) and lower VacLok for immobilization. At this time, patients that may be suitable for an HDR-BT boost underwent a volume study. The patient was scanned in the lithotomy position; the radiation oncologist subsequently reviewed the volume study’s axial images to assess for potential pubic arch interference (PAI) (Figure 1). If no PAI or median lobe hypertrophy (MLH) was detected, a BT boost was planned for prior to EBRT, to ensure that implantation can be completed with no delays.

Brachytherapy procedure

Patients were asked to perform an enema on the day of the procedure. Upon admission, each patient was brought to the operating room and placed in the lithotomy position under general anesthesia. A rectal exam was performed. A wet towel was used to move the genitalia superiorly, exposing the perineum, which was then shaved and prepped as a sterile field. A Foley balloon with contrast was inserted into the bladder to assist intra-operatively and for treatment planning. After placing the transducer condom holding ultrasound gel over the ultrasound probe, the transrectal ultrasound (TRUS) was mounted to the stepper unit and inserted into the rectum for image guidance. A template was then attached to the ultrasound stepper. The TRUS probe was adjusted such that the posterior border of the prostate/seminal vesicle (SV) was parallel to the horizontal axis of the ultrasound display, with the probe exerting mild pressure against the anterior rectal wall. At mid-gland between the base and apex of the prostate (axial view), the prostate/SV was centered on the ultrasound display, with the “D-row” of the template aligned with the Foley catheter. Seventeen-gauge titanium steel interstitial needles (250 mm) with trocar points (Varian Medical Systems, Palo Alto, CA) were implanted using the template and TRUS image guidance. Needles are inserted anterior to posterior to minimize TRUS artifact from prior needles. Peripheral needles and four central, periurethral needles were typically sufficient to cover the prostate and SV.

Following postoperative recovery, the patient was transferred in the supine position to the clinic for CT simulation. At the attending physician’s discretion, minor adjustments to needle depth could be made at the time of the simulation. A Zephyr HDR patient positioning and transfer system (Orfit Industries NV, Wijnegem, Belgium) was used to transfer the patient with minimal motion from CT simulation to the HDR treatment bunker to assure each patient was treated in the planned position.

**Figure 2:**
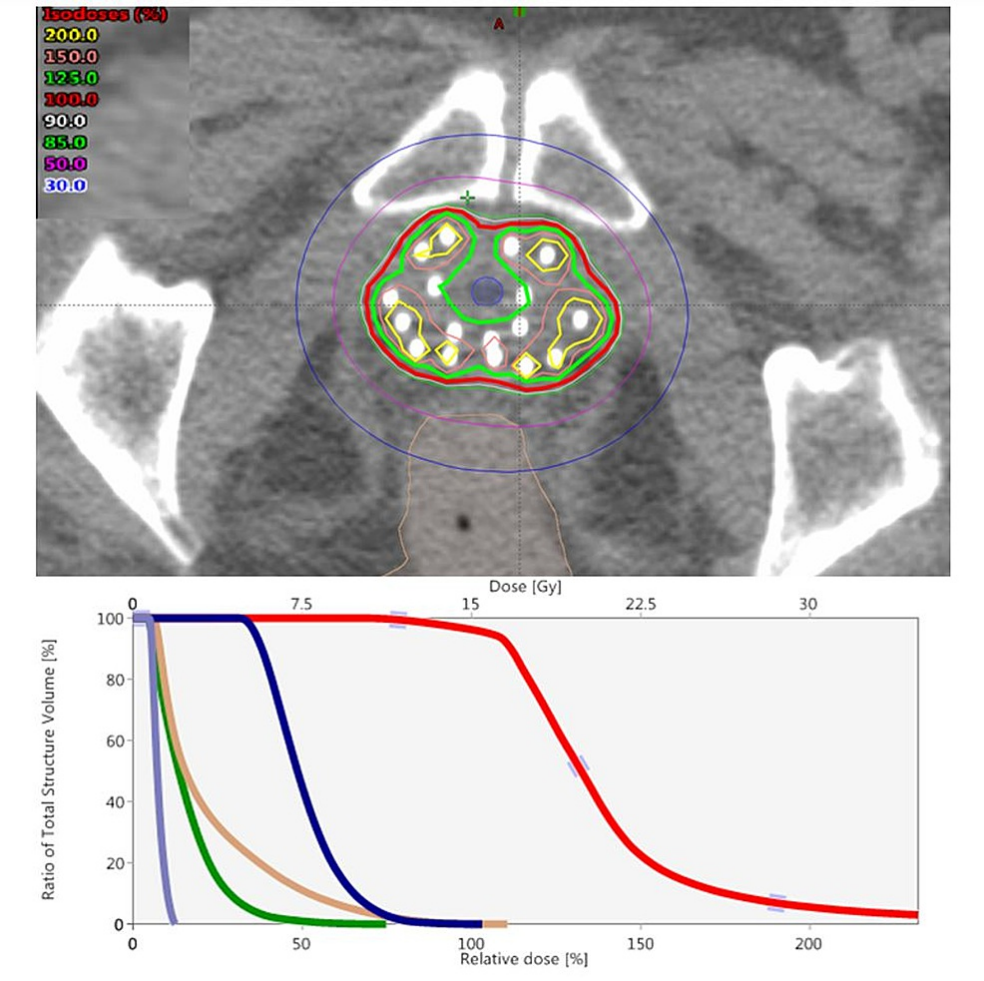
Top pane: Representative implantation of a patient with high-risk prostate cancer, 29 cc gland, 17 needle catheters used. Bottom pane: Implant dose-volume histogram

A radiation oncologist contoured the CTV and avoidance structures (rectum, bladder, sigmoid, urethra, small bowel, urogenital diaphragm (UGD), and bladder neck). MRI pelvis (if available) was fused to CT simulation and used to ensure coverage of any dominant lesion in the prostate. Each needle was digitized and given an available active length for source dwell positions corresponding to the length of the target structure in relation to the needle. A plan was then optimized using the linear source model, TG-43 based, Nelder-Mead Simplex algorithm in the Varian BrachyVision (Varian Medical Systems) treatment planning system. Inverse planning followed by dose-shaping was employed for plan optimization with goals based on desired target coverage, limits to target heterogeneity, and dose tolerances to surrounding critical structures. Our dose and fractionation were 15 Gy x 1 fraction for boost [10] and 13.5 Gy x 2 for monotherapy [11]. The dose constraints and planning aims used by our department are listed in Table 1. See Figure 2 for a typical implant.

**Table 1:** Target planning aims and dose constraints Rx - prescription dose, PTV - planning target volume, CTV - clinical target volume, ECE - extracapsular extension, SVI - seminal vesicle invasion, GUD - genitourinary diaphragm

Rx: 15 Gy x 1 fraction to PTV		
(PTV = CTV = Prostate/ECE/SVI)		
	V100% > 90-95%	-Dwell times outside PTV minimized; “Horseshoe shape” of the 125% isodose
	V125% < 55-60%	
	V150% < 25-30%	
Urethra	V118% < 0.1cc	-Avoid hotspot at urethra below the prostate apex (GUD) – highest risk of stricture
	V115% < 1-5%	
	V100% < 85-90%	
Bladder and rectum	V75% < 1.0cc	
Bladder neck	80-85% max	

Treatment was performed using the VariSource iX HDR Brachytherapy Afterloader (Varian Medical Systems) with a single, 5 mm Iridium-192 source with 5 mm dwell position spacing. The treatment lasted between 10 and 30 min depending on the source activity and the number of needles. The device was then removed in the clinic, hemostasis was achieved, and the patient was discharged with Tylenol #3 PRN and phenazopyridine 97.5 mg PRN, Bactrim DS PO BID for 7 days, and tamsulosin 0.4 mg PO daily to reduce pain, infection, and urinary obstruction, respectively. Nurses contacted the patient the following day to screen for any concerning post-operative symptoms.

Data analysis

Institutional Review Board IRB approval was granted (Protocol Name: The Use of Brachytherapy Boost in Prostate Cancer, Number: H-45492). Subsequently, we conducted a retrospective, single-institution review of patients with prostate cancer treated with an HDR-BT boost or monotherapy. All men with prostate cancer treated between July 1, 2016, and December 31, 2019, were identified from existing clinical rosters. Clinical demographics, dosimetric and clinical details of treatment, and outcomes were abstracted for all subjects. Data are presented in tabular format with descriptive statistics.

## Results

Outcomes

Since the inception of our prostate HDR program, 17 patients have been treated for a total of 18 implants (there was one brachytherapy monotherapy for favorable intermediate-risk consisting of two implants). See Table 2 for patient characteristics. No implant was aborted. There was no acute urinary retention. The median initial prostate-specific antigen (iPSA) was 20.1 ng/mL, median post-treatment PSA was 0.35 ng/mL at a median follow-up of 28.5 months. There was no change in median AUA score (pre-treatment and post-treatment both 9). There was no change in the average quality-of-life score (pre-treatment and post-treatment both 2 = mostly satisfied). Median SHIM change not reported due to variability in ADT duration and completion at most recent follow-up. 100% of patients received ADT. There was one biochemical failure occurring in a very high-risk patient (iPSA 45 ng/mL, GL 4+5=9, 11 of 12 cores positive) at 37 months after treatment; PSA now controlled on abiraterone, leuprolide, and prednisone. Our biochemical recurrence-free survival (b-RFS) is therefore 94% at a median follow-up of 28.5 months (range 8.2-50 months). Duration of treatment delivery reduced each year (2016 = 11.4 hrs, 2019 = 8.7 hrs). One limitation to reducing treatment times was OR-based implant and clinic-based treatment planning and delivery, necessitating lengthy PACU clearance and transport times. There was no reported urethral stricture within the follow-up period.

**Table 2:** Patient characteristics and outcomes TRUS - Transrectal ultrasound; GL - Gleason score; iPSA - initial prostate-specific antigen; ND - no data

	ID	Clinical T Stage	TRUS (gm)	Primary GL Grade	Secondary GL Grade	Sum GL	% Cores Positive	iPSA	Current PSA	ΔAUA	ΔQoL
Favorable	3	T2b	21	3	4	7	33%	6.05	0.23	1	2
Intermediate	9	T2a	30	3	4	7	42%	3.46	0.20	0	0
	14	T1c	39	3	4	7	25%	6.00	0.20	1	-1
Unfavorable	2	T1c	55	4	3	7	8%	4.61	0.37	-1	0
Intermediate	4	T1c	32	4	3	7	42%	6.12	0.58	1	3
	10	T1c	36	4	3	7	50%	15.30	0.00	3	0
	13	T1c		3	4	7	50%	17.80	0.02	0	-1
	16	T1c	38	3	4	7	67%	8.85	0.37	4	0
	17	T1c	22	3	4	7	33%	13.60	0.30	ND	ND
High	5	T3a	30	3	4	7	25%	37.60	0.00	-4	3
	6	T1c	39	4	4	8	33%	10.99	0.13	2	0
	11	T2a	37	4	3	7	42%	31.90	0.00	4	-2
	15	T1c	29	4	4	8	25%	10.50	0.00	1	0
Very High	1	T2c	32	4	5	9	92%	45.19	3.40	6	0
	7	T2c	40	4	4	8	100%	58.70	0.06	-10	0
	8	T1c	41	5	5	10	33%	11.50	0.11	0	-1
	12	T1c	59	4	4	8	50%	53.90	0.00	0	0

Dosimetry

In our cohort, the median (IQR) prostate coverage is as follows: V100 (%) was 96.4 (1.9), V125 (%) was 56.6 (10.6), and V150 (%) was 26.6 (3.4), therefore, all parameters for target coverage are acceptable according to Table 1. For organs-at-risk parameters, the median (IQR) dose to the urethra is as follows: V115 (%) was 3.5 (7.9) and V100 was 56.5 (27.3). Therefore, the target aims for this important structure were met per Table 1 guidelines. Bladder V75% (cc) was 0.3 (0.9) and rectum V75% (cc) was 0.9 (0.4), again median (IQR), which are also in agreement with guidelines. Finally, the median (IQR) maximum dose to the bladder neck (Dmax) was 13.4 Gy (1.4 Gy), which is slightly above the guideline maximum of 85% of the Rx dose (which would be 12.75 Gy), indicating a deviation from planning aims. Table 3 lists all tracked dosimetric parameters for our patient cohort.

**Table 3:** Dosimetric parameters IQR - interquartile range

	ID	ProstateV100 (%)	Prostate V125 (%)	Prostate V150 (%)	UrethraV125 (%)	Urethra V118% (cc)	Urethra V115 (%)	Urethra V100 (%)	Bladder V75% (cc)	Bladder Neck Dmax (Gy)	GUD 0.01cc (Gy)	GUD max (Gy)	GUD V110% (cc)	Rectum V75% (cc)
Fav Int	3	91.3	53.0	26.0	0.0	0.0	1.8	33.1	0.2	12.2	16.3	18.0	0.0	0.9
	9	97.4	56.5	31.7	0.0	0.1	10.4	34.4	1.3	13.9	10.0	10.8	0.0	0.3
	14	96.6	65.6	27.3	0.0	0.1	14.5	36.9	0.0	8.8	13.7	19.9	0.0	0.4
		95.5	55.5	26.0	0.0	0.0	5.7	50.9	0.0	9.7	10.3	13.5	0.0	1.0
Unfav Int	2	95.1	51.0	25.1	0.0	0.0	0.5	59.5	0.8	12.7	11.4	14.2	0.0	0.8
	4	95.5	51.9	27.2	0.0	0.0	1.6	84.6	0.9	13.8	15.0	16.5	0.0	1.1
	10	97.0	66.3	35.7	0.1	0.2	18.1	36.7	0.0	13.7	12.3	13.5	0.0	0.1
	13	99.1	51.6	22.5	0.0	0.0	7.2	65.7	0.0	13.3	16.1	27.3	0.0	1.1
	16	96.6	68.6	22.5	0.0	17.7	25.7	53.5	0.0	15.9	9.6	10.3	0.0	0.6
	17	94.0	62.0	23.6	0.0	0.0	0.7	60.0	0.0	11.3	18.1	20.8	0.0	1.3
High	5	93.7	59.8	26.5	0.0	0.0	1.7	48.9	1.4	11.6	16.1	17.2	0.0	1.0
	6	97.1	56.6	28.1	0.0	0.0	2.9	62.8	0.4	13.5	6.6	6.9	0.0	1.0
	11	98.2	51.6	28.2	0.0	0.0	3.6	79.8	1.1	13.8	17.3	17.3	0.0	1.0
	15	96.3	63.9	22.5	0.0	0.1	6.3	80.3	0.0	13.2	12.7	15.6	0.0	2.5
Very High	1	95.0	47.7	24.3	0.0	0.0	3.4	40.9	0.9	14.5	13.7	17.1	0.0	0.6
	7	95.5	62.3	27.3	0.0	0.0	0.6	69.0	0.7	13.2	16.9	21.0	0.0	0.9
	8	96.4	50.0	30.3	0.0	0.0	1.7	37.0	1.1	14.3	16.2	22.6	0.0	0.7
	12	99.1	57.1	26.7	0.0	0.1	12.4	64.1	0.0	14.7	16.2	19.3	0.0	0.1
Median		96.4	56.6	26.6	0.0	0.0	3.5	56.5	0.3	13.4	14.3	17.2	0.0	0.9
IQR		1.9	10.6	3.4	0.0	0.0	7.9	27.3	0.9	1.5	4.6	6.1	0.0	0.4

## Discussion

Evidence supporting the use of brachytherapy

There is a robust pool of evidence demonstrating the superiority of LDR BT boost (Phase III) and the feasibility and efficacy of the HDR boost (2% rate of local failure at both five and 10 years, Phase II) when compared to EBRT alone [3-4,12]. In a randomized phase III trial of 218 patients assigned to EBRT alone or EBRT with HDR-BT boost, Hoskin et al. showed significantly better relapse-free survival (RFS) in the group receiving HDR-BT when compared to EBRT alone (median time to relapse: 116 months vs. 74 months, p=0.04) [13]. In a retrospective cohort study of 1809 men with Gleason 9-10 prostate cancer treated with either radical prostatectomy, EBRT alone, or EBRT with HDR-BT boost, Kishan et al. showed marked improvement in five-year prostate cancer-specific mortality (PCSM) and five-year rate of distant metastases in the HDR-BT boost arm (3% and 8%) compared to radical prostatectomy (12% and 24%) and EBRT alone (13% and 24%) [14].

The rates of biochemical failure, are, in fact, magnified by using a surgical PSA definition (0.2-0.4 ng/mL) when compared to Phoenix definition (nadir + 2 ng/mL): the seven-year b-PFS after dose-escalated EBRT declined from 76% using nadir + 2 ng/mL to 38% using the > 0.2 ng/mL threshold (p > 0.001), whereas the BT boost arm revealed no significant difference using the two definitions (both >85%, p=0.3) [15].

Improvements in disease control are expected in BT boost given the superior dosimetric characteristics. Its inherent dose heterogeneity allows for the targeted deposition of the dose to areas of high disease risk while ensuring that volumes outside the prostate receive minimal radiation. BT has a potential role in the treatment of prostate cancers across all risk categories. In high-risk prostate cancer, HDR-BT boost combines the advantages of EBRT to cover the pelvis, which is often considered for elective irradiation given the high risk of nodal involvement, with the highly conformal, high-dose boost delivered to the prostate by HDR-BT [16].

Furthermore, the incidence of toxicities or adverse events is acceptable; a phase II clinical trial, RTOG 0321, reported <5% grade 3 or greater late toxicities and GI/GU adverse events in patients treated with EBRT and HDR-BT boost [12,17].

Determining fractionation/dose

There has been an interest in multi-fraction HDR-BT as monotherapy for the treatment of low-intermediate-risk prostate cancer (i.e. no EBRT). Hauswald et al. report 97.8% actuarial 10-year PSA PFS with local control rates of 99.7% in 448 men with low-intermediate risk prostate cancer treated with HDR-BT monotherapy, 43.5 Gy in six fractions [18]. Given the challenges logistically and the resource ramifications of multiple implantations for HDR-BT, there has been great interest in reducing the number of fractions needed. Per biologically equivalence dose (BED) calculations, dose fractionations of 11.5 Gy × 3, 13.5 Gy × 2, or 19 Gy × 1 would deliver BED of 260-280 Gy, which would be theoretically equivalent to EBRT of 110-120 Gy at 2 Gy per fraction (based on an alpha/beta of 1.5) [11]. Hoskin et al. show promising results treating intermediate and high-risk prostate cancers with HDR-BT as monotherapy in two or three fractions with four-year freedom from biochemical relapse rates of 77% and 81%, respectively, and relapse-free survival of 68% and 79%, respectively [19]. Notably, the patient profile in this series of 293 patients was exclusively intermediate and high-risk. Data from Morton et al. support the use of two-fraction brachy-monotherapy in low-intermediate-risk prostate cancer, albeit with only five-year follow-up data. Single-fraction HDR-BT for the treatment of low-intermediate-risk prostate cancer would be convenient, as it would mirror LDR-BT; unfortunately, according to this same cohort of data, prostate cancer control rates are unacceptably low [20]. This regimen was also attempted by Siddiqui et al. who treated 68 low-intermediate-risk prostate patients. Five-year follow-up data revealed higher-than-expected rates of biochemical and local failure, as well as biopsy-proven failure in 18.8% of patients at four years [21]. Further, a trial utilizing an MRI-guided focal dominant intraprostatic lesion (DIL) boost to 23 Gy showed similar inferiority [22].

Among suitable candidates, prostate BT has proven to be the optimal modality for prostate cancer control with a favorable side effect profile [3,23]. Our data reveal a b-RFS of 94% at over two years later, without acute or late grade 3 or higher toxicity. Notably, our one patient who did fail therapy did so at 37 months and was initially a very high-risk prostate cancer.

Deciding between HDR and LDR, and image-guidance

One of LDR’s greatest advantages is that it can be delivered as a single outpatient procedure under anesthesia, whereas HDR requires multiple fractions and possibly overnight hospitalization. However, it must be remembered that while, initially, HDR was delivered over several fractions (i.e. 4-9) [24-25], the trend has been for fewer, larger fractions [26]; with the caveat that the holy grail, a single 19 Gy (23 Gy boost to DIL) implant (for monotherapy) had higher-than-expected rates of biochemical failure, and therefore the evidence supports the use of at least two implants for HDR monotherapy [21-22]. Whereas, a single 15 Gy has become the standard HDR boost dose in current Radiation Therapy Oncology Group clinical trials (RTOG 0924 and RTOG 1115).

With real-time ultrasound-based planning in the OR, HDR implants can be completed in approximately two hours [27], whereas CT-based planning can take approximately four hours depending on department experience. Our department’s treatment times (approximately eight hours) are within the limitation that our OR is separate from our radiation facility, and therefore our patients require clearance from the post-anesthesia care unit (PACU) and transportation to our clinic prior to CT simulation and further treatment planning. In the future, utilizing bilateral pudendal block to obviate going to the OR and awaiting transportation may be a great option to boost efficiency in our department. This has been successfully done in Germany [28], as well as suggested in recent brachytherapy literature as a strategy to mitigate patient exposures to COVID-19 [29].

A high dose rate delivers treatment over 10-30 minutes using a high activity Ir-192 source (final treatment time depends on the number of catheters used, the dwell time, and the activity of the source). In the modern era, LDR delivery has been a TRUS-based, single outpatient procedure.

At the conclusion of HDR treatment, no radioactivity remains in the patient, in contrast to LDR. Because the seeds are “live” in LDR, treatment cannot be aborted in the case of poor or complicated implantations. Furthermore, the misadministration of a single seed or whole strand is a difficult situation to correct. In this sense, HDR can be thought of as more forgiving, and less operator-dependent; a poorly placed needle can be removed or not used at planning, and dose optimization within dwell positions can create tailored plans from patient to patient or fraction to fraction. Taking advantage of dwell times and different treatment catheters, the dose of HDR can be carefully sculpted around OARs while the dose can be escalated in dominant sites of disease or areas of extracapsular extension using MRI fusion at the time of CT-based planning.

Another consideration is the situation with T3 disease (extracapsular extension and/or seminal vesical invasion). This risk factor is an important consideration, as HDR (because it is delivered via a temporary, enclosed catheter) has the potential for improved dose coverage in this disease; whereas LDR is commonly prescribed only up to 3 mm outside the capsule, and therefore significant extracapsular extension or extensive seminal vesicle invasion (SVI) coverage may be limited.

Yet another consideration is the logistics of treatment. For an HDR-BT boost, generally, the fraction is delivered prior to the external beam to ensure successful treatment prior to initiating the external beam (i.e. to prevent a situation wherein unforeseen pubic arch interference causes treatment break). We generally waited one month following the implant to start the external beam. In contrast, oftentimes, an LDR boost is delivered after completing the external beam and therefore could theoretically cause an unplanned treatment break should there be any difficulty with the implant.

From a patient perspective, HDR-BT allows for more post-procedure freedom at home. The lack of an active radioactive source in the patient obviates the need to avoid infants and pregnant females. Further, condom use is unnecessary after HDR-BT. Data suggest that HDR-BT is associated with less acute urinary toxicity than LDR BT although both modalities have similar late urinary toxicity rates [30]. HDR-BT also appears to result in less erectile dysfunction [31].

From a practical perspective, while a radiation oncologist can be trained to do LDR-BT over the course of a few months, when they return to practice, if they initially do not have a high volume of referrals (many practices have less than 12 cases per year [32-33], they may quickly lose confidence in performing the procedure and decide to no longer offer this treatment to their patients.

Finally, a single large-fraction HDR has been shown to induce transcriptional changes in the tumor genome, which may enhance radiation sensitivity. Therefore, subsequent radiation with an external beam may theoretically be rendered more effective; however, this premise requires additional study [34].

Ultimately our department chose to pursue an HDR-based program after considering the above factors, and as we already have a high volume GYN BT program and afterloader with BT suite available, much of the initial investment in equipment was obviated.

One of the limitations of the present study includes the lack of long-term follow-up data; the advantages of a BT boost over EBRT alone bear out more significantly in more mature data sets. Another potential limitation to our present study is our department already has a busy GYN HDR practice and therefore the initial investment in an HDR afterloader and treatment bunker was obviated. Likewise, our brachytherapists are already experienced with interstitial type brachytherapy procedures, therefore, the adoption of this procedure was likely smoother than average from a technical standpoint. Finally, another limitation to our study was the time intensity of the procedure owing to the fact that our OR is separate from the radiation facility, necessitating lengthy PACU and transport times. Once familiar with the technique, our procedures took about eight hours from patient hospital admission to discharge from the radiation facility. While not all steps of the procedure require direct radiation oncologist supervision, it could certainly be cumbersome for an individual staff radiation oncologist without a trainee’s assistance.

## Conclusions

Our department was able to implement an OR-based HDR prostate BT program with CT-based planning within our cancer center. Dosimetric data were acceptable, and patients had effective treatments. There was no grade 3 toxicity to report. A BT boost represents the optimal modality in well-selected, organ-confined, intermediate- and high-risk prostate cancers. Monotherapy for low-intermediate-risk prostate cancer is a compelling treatment option to which we add to the growing evidence. This paper is meant to be a guide for residency programs and community practices wanting to start a CT-based HDR prostate BT program to make this modality more available to patients with aggressive localized prostate cancers, as well as increase radiation oncology trainee familiarity and comfort with this underutilized modality.
